# Clinical Routine FDG-PET Imaging of Suspected Progressive Supranuclear Palsy and Corticobasal Degeneration: A Gatekeeper for Subsequent Tau-PET Imaging?

**DOI:** 10.3389/fneur.2018.00483

**Published:** 2018-06-20

**Authors:** Leonie Beyer, Johanna Meyer-Wilmes, Sonja Schönecker, Jonas Schnabel, Eva Brendel, Catharina Prix, Georg Nübling, Marcus Unterrainer, Nathalie L. Albert, Oliver Pogarell, Robert Perneczky, Cihan Catak, Katharina Bürger, Peter Bartenstein, Kai Bötzel, Johannes Levin, Axel Rominger, Matthias Brendel

**Affiliations:** ^1^Department of Nuclear Medicine, University Hospital, LMU Munich, Munich, Germany; ^2^Department of Neurology, University Hospital, LMU Munich, Munich, Germany; ^3^Department of Psychiatry, University of Munich, Munich, Germany; ^4^German Center for Neurodegenerative Diseases (DZNE), Munich, Germany; ^5^Neuroepidemiology and Ageing Research Unit, School of Public Health, Imperial College, London, United Kingdom; ^6^West London Mental Health NHS Trust, London, United Kingdom; ^7^Institute for Stroke and Dementia Research, University of Munich, Munich, Germany; ^8^Munich Cluster for Systems Neurology (SyNergy), Munich, Germany; ^9^Department of Nuclear Medicine, Inselspital, University Hospital Bern, Bern, Switzerland

**Keywords:** atypical parkinsonian syndrome, progressive supranuclear palsy, corticobasal degeneration, F-18-FDG, PET, clinical routine

## Abstract

**Background:** F-18-fluordeoxyglucose positron emission tomography (FDG-PET) is widely used for discriminative diagnosis of tau-positive atypical parkinsonian syndromes (T+APS). This approach now stands to be augmented with more specific tau tracers. Therefore, we retrospectively analyzed a large clinical routine dataset of FDG-PET images for evaluation of the strengths and limitations of stand-alone FDG-PET.

**Methods:** A total of 117 patients (age 68.4 ± 11.1 y) underwent an FDG-PET exam. Patients were followed clinically for a minimum of one year and their final clinical diagnosis was recorded. FDG-PET was rated visually (positive/negative) and categorized as high, moderate or low likelihood of T+APS and other neurodegenerative disorders. We then calculated positive and negative predictive values (PPV/NPV) of FDG-PET readings for the different subgroups relative to their final clinical diagnosis.

**Results:** Suspected diagnoses were confirmed by clinical follow-up (≥1 y) for 62 out of 117 (53%) patients. PPV was excellent when FDG-PET indicated a high likelihood of T+APS in combination with low to moderate likelihood of another neurodegenerative disorder. PPV was distinctly lower when FDG-PET indicated only a moderate likelihood of T+APS or when there was deemed equal likelihood of other neurodegenerative disorder. NPV of FDG-PET with a low likelihood for T+APS was high.

**Conclusions:** FDG-PET has high value in clinical routine evaluation of suspected T+APS, gaining satisfactory differential diagnosis in two thirds of the patients. One third of patients would potentially profit from further evaluation by more specific radioligands, with FDG-PET serving gatekeeper function for the more expensive methods.

## Introduction

Atypical parkinsonism refers to a group of neurodegenerative syndromes presenting symptoms resembling those of Parkinson's disease (PD), but generally with earlier onset, faster progression, and poor response to dopamine replacement treatments. Chief among the atypical parkinsonian syndromes are progressive supranuclear palsy (PSP) and corticobasal syndrome (CBS), which both represent tauopathies. Whereas PSP predominantly presents with a predominantly supranuclear vertical gaze palsy and early postural instability with falls, CBS typically manifests as markedly asymmetrical parkinsonism in conjunction with apraxia or cortical sensory disturbance (1). The incidence of atypical parkinsonism syndromes is low relative to PD (2), but their more aggressive course calls for improved discriminative diagnosis methods and disease specific therapies, such as anti-tau regimens for PSP and CBS.

The past decade has seen extensive investigations of brain glucose metabolism by F-18-fluorodeoxyglucose positron-emission-tomography (FDG-PET) in PSP and CBS patients (3–8), aiming to detect disease-specific patterns of altered glucose consumption. Significant reductions of FDG uptake were observed in PSP subjects in the bilateral anterior cingulate gyrus and the midbrain (6). In CBS subjects, glucose hypometabolism was found in the central region, the frontal and parietal association areas, the putamen contralateral to the clinically affected side and the bilateral thalamus (7).

Until recently, molecular imaging of glucose metabolism was not considered by diagnostic (9, 10), even though FDG-PET is often requested to determine the presence of neurodegeneration in suspected PSP and corticobasal degeneration (CBD) as the major subgroup of CBS; however recently proposed diagnostic criteria now include imaging findings by FDG-PET for PSP (11). The importance of FDG-PET is underlined by the fact that the correlation between the clinical diagnostic criteria and a neuropathological confirmation is still insufficient in suspected PSP and CBD (12, 13).

Most of the mentioned molecular imaging studies were performed in academic settings with highly characterized patient populations to evaluate the FDG uptake patterns, sometimes with machine learning algorithms. Sensitivity, specificity, positive and negative predictive values for differential diagnosis of different parkinsonian syndromes were consistently > 80% in a recent test of an automated image-derived classification procedure (14). In clinical routine of a nuclear medicine department, the demands for differential diagnosis of suspected atypical parkinsonism by FDG-PET is often more complicated, and hindered by the less extensive clinical information, a broad spectrum of possible diagnoses, and missing follow-up data. Besides the high sensitivity for concomitant dementia in parkinsonian syndromes even in clinical routine, FDG-PET imparts inherently unspecific information about synaptic dysfunction, which is the common thread of all neurodegenerative disorders (15). More recent developments in molecular imaging for discriminative diagnosis of PD, atypical parkinsonism, and neurodegenerative diseases in general focus on disease-specific neuroreceptor changes (16) and neuropathological hallmarks (17, 18).

The advent of tau-PET imaging presents new prospects for identification of PSP and CBD with the pyridoindole F-18-AV1451 (19, 20) and arylquinoline F-18-THK5351 (21, 22) and their derivatives. In nuclear medicine clinical routine it would be critical to establish suitable diagnostic algorithms guiding the decision to augment an established and relatively inexpensive tracer (FDG) with a less conventional and more expensive tau-PET investigation; the higher cost must be justified by the additive diagnostic value. Therefore, we made a retrospective analysis of clinical routine FDG-PET data obtained during a 7-year period for complex cases referred to a tertiary center with suspected tau-positive atypical parkinsonism. Our objective was to determine if FDG-PET findings, in the light of follow-up to a definite diagnosis, can predict cases where additional tau-PET examination would potentially have resolved the initial diagnostic uncertainty. By making this identification of patient groups we aimed to create a basis for future prospective treatment trials.

## Materials and methods

### Study design and patient enrollment

All participants were examined at the Departments of Neurology and Psychiatry, respectively, and Institute for Stroke and Dementia Research, and were scanned in a clinical routine setting at the Department of Nuclear Medicine between 2009 and 2015. After undergoing cognitive testing and partially assessments of nigrostriatal innervation by DaTSCAN-SPECT, structural MRI imaging and CSF sampling, patients with a differential diagnosis of suspected PSP/CBD were referred for FDG-PET examination. To avoid a selection bias, all patients with suspected PSP/CBD in the observation period were considered as a primary sample (*n* = 151). Inclusion criteria were defined by at least one year of clinical follow-up (*n* = 122) and successful FDG-PET imaging scan (i.e., no early termination by the patient or not correctable head motion). Exclusion criteria were earlier events of stroke (*n* = 3) or current severe affective disorders (*n* = 2), which might influence FDG-PET pattern or clinical features. A final clinical diagnosis was recorded at clinical follow-up, based on current diagnostic criteria of parkinsonian syndromes and other neurodegenerative disorders. One hundred and seventeen patients with suspected PSP/CBD were eligible for the final analysis.

### Clinical assessments, MRI and CSF

The majority of patients (61%; 71/117) received neuropsychological testing including cognitive evaluation at the time of the FDG-PET scan [Mini-Mental-State Examination (MMSE)]. Years of education were recorded and a neurological examination was performed. Symptom categories for clinical classification of PSP and CBS were assessed binarized (as Yes/No) at the time of the FDG-PET scan. Patients with suspected PSP were classified for probable or possible PSP according to NINDS-SPSP criteria (10), as the observation period of this retrospective analysis took place before publication of newest PSP criteria (11). Patients with suspected CBD were classified for probable or possible CBS according to criteria defined by Armstrong et al. (9). The Supplementary Material gives an overview on symptom categories and their necessary composition for the establishment of a clinical diagnosis.

Structural MRI (*n* = 96) and CSF samples (*n* = 97) were available from >80% of the 117 patients, whereas DaTSCAN was performed in less than half of the patients (*n* = 49; 42%).

### PET imaging

#### FDG PET acquisition

FDG-PET images were acquired on a GE Discovery 690 PET/CT scanner or a Siemens ECAT EXACT HR+ PET scanner. All patients had fasted for at least 6 h, and had a maximum plasma glucose level of 150 mg/dl at time of scanning. A single intravenous dose of 140 ± 7 MBq FDG was administered while the patients rested in a room with dimmed light and low noise level, where they remained undisturbed for 20 min. After positioning in the scanner, a series of three static emission frames of 5 min each was acquired from 30 to 45 min p.i. on the GE Discovery 690 PET/CT, or from 30 to 60 min p.i. on the Siemens ECAT EXACT HR+ tomograph. A low-dose CT scan or a transmission scan with external ^68^Ge-source performed just prior to the static acquisition was used for attenuation correction. PET data were reconstructed iteratively (GE Discovery 690 PET/CT) or with filtered-back-projection (Siemens ECAT EXACT HR+ PET). After correction for movement between frames, the static scans were averaged.

#### Visual analysis of FDG-PET

**Figure 1:**
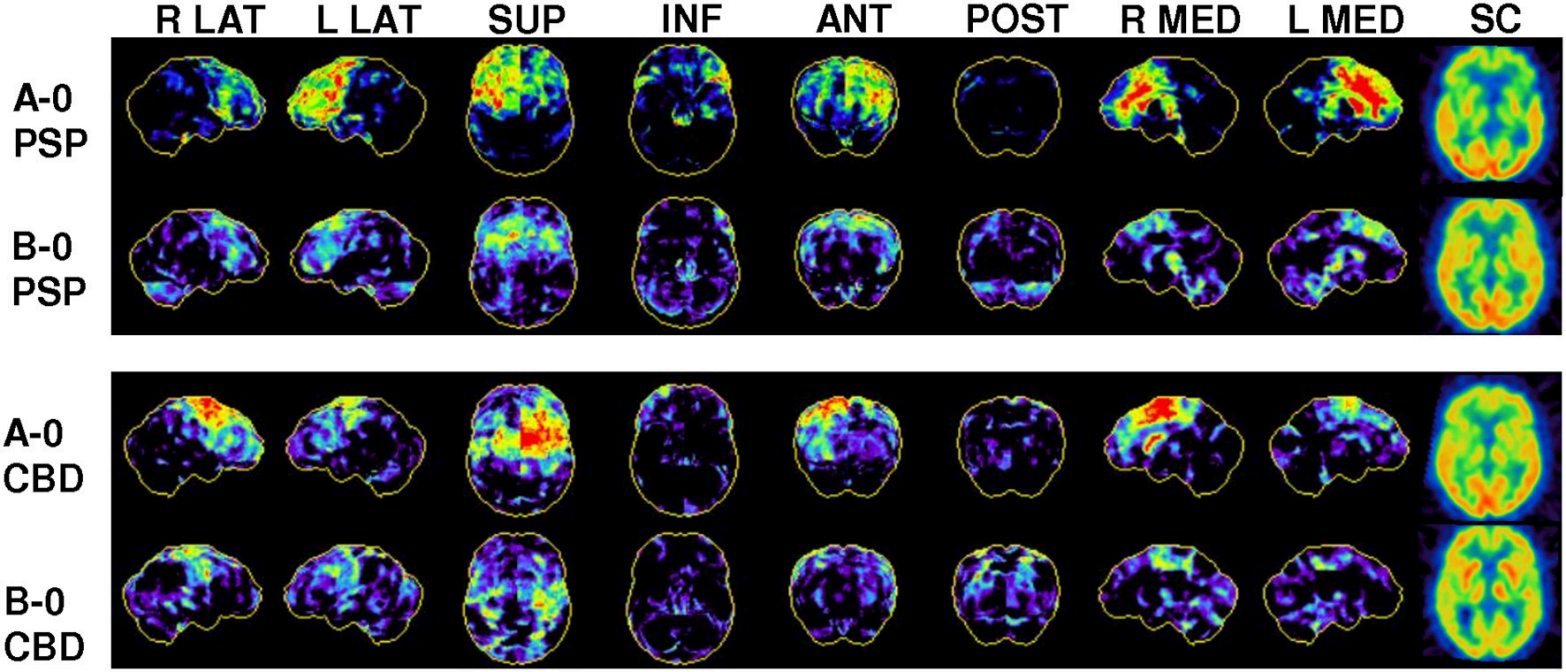
Representative FDG-PET images of study subgrous: Three-dimensional stereotactic surface projections (3D-SSP) of normalized FDG uptake from right/left lateral (R LAT/L LAT), superior (SUP), inferior (INF), anterior (ANT), posterior (POST), right/left medial (R MED/L MED) views and a subcortical (SC) section image in the axial pain for four out of nine defined subgroups according to their likelihood for PSP/CBD. In PSP subjects, asymmetrical or bilateral hypometabolism was found predominantly in the prefrontal cortices, the anterior cingulate gyrus, and the midbrain. In CBS, asymmetrical hypometabolism was observed in the central region, the putamen and thalamus.

For visual image interpretation of FDG-PET images, three-dimensional stereotactic surface projections (3D-SSP) (23) were generated using the software Neurostat (Department of Radiology, University of Washington, Seattle, WA, U.S.A.). A senior expert in Nuclear Medicine visually assessed axial and sagittal slices and the 3D-SSP images depicted as normalized tracer uptake and Z-score maps against reference images from a group of age-matched healthy controls. The reader had access to clinical information (available in all cases) and, when available, DaTSCAN and/or structural MRI information. Based on the anatomic pattern of hypometabolism the FDG-PET images were first rated for likelihood (high = A/moderate = B/low = C likelihood) of a neurodegenerative metabolism pattern typical for PSP or CBD (24). Next, the FDG-PET scan was rated for likelihood (high = 2/moderate = 1/low = 0) of a metabolism pattern typical of a neurodegenerative condition other than PSP or CBD (mainly Alzheimer's disease, PD, frontotemporal lobar degeneration, multiple system atrophy). Based on permutations of the FDG-PET findings we defined nine possible subgroups (see Table 1 and Figure 1).

**Table 1:** Subgroup definition by FDG-PET findings according to the likelihood for PSP/CBD (A-C) and other neurodegenerative diseases (0-2).

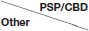	**High**	**Moderate**	**Low**
Low	A-0	B-0	C-0
Moderate	A-1	B-1	C-1
High	A-2	B-2	C-2

Additionally the reader had to assert positivity or negativity for PSP/CBD as a binarized read-out. A likelihood for PSP/CBD exceeding the likelihood for other neurodegenerative diseases was defined as positive in this regard, whereas equal or lower likelihoods in the same comparison were defined as negative.

### Statistical analysis

Normality of data distribution was assessed by the Kolmogorov-Smirnov test. The nonparametric χ^2^ test was used to test for significantly differing probability of trichotomous likelihood of disease-typical FDG-PET patterns (high, medium, low) among subgroups. The diagnostic accuracy was tested by calculating the sensitivity, specificity and positive/negative predictive value (PPV/NPV) using the final clinical diagnosis after ≥ 1 year of follow-up as the truth. We did not perform an a priori sample size calculation. A significance level of *p* < 0.05 for rejection of the null hypothesis was applied in all analyses. All statistical tests were performed using SPSS (version 23.0, IBM, Chicago, IL).

## Results

### Demographics

A total of 117 subjects (51% male) were included in the study (Table 2). Fifty-three patients (34% male) had been referred with suspected PSP and 64 patients (52% male) with suspected CBD. 16 patients were referred with both possible differential diagnoses, and were assigned to one of the two aforementioned subgroups according to their more likely diagnosis. The mean age was 68.4 years (SD ± 11.1). The clinical diagnosis was confirmed by clinical follow-up ≥ 1 year in 31/53 (59%) patients with suspected PSP and in 31/64 (48%) patients with suspected CBD. Additional findings from neuropsychological testing and a summary of presence or absence of single symptom categories are provided in the Supplemental Results.

**Table 2:** Demographics and composition of the study collective.

	***N***	**Final diagnosis PSP/CBS**	**Age (y)**	**Gender (♂, male, ♀, female)**	**Education (y)**	**MMSE**
All	117	62 (53%)	68.4 ± 11.1	51 ♂ 56 ♀	13.8 ± 3.6	25.5 ± 4.2
PSP	53	31 (59%)	72.3 ± 8.0	27 ♂ 26 ♀	14.2 ± 3.5	24.8 ± 4.3
Probable	22	15 (68%)	70.7 ± 7.7	11 ♂ 11 ♀	13.8 ± 2.5	25.1 ± 3.6
Possible	23	14 (61%)	71.9 ± 7.8	12 ♂ 11 ♀	14.3 ± 4.5	24.2 ± 4.9
Criteria not fulfilled	8	2 (25%)	77.9 ± 7.8	4 ♂ 4 ♀	15.0 ± 3.6	25.5 ± 5.8
CBS	64	31 (48%)	65.2 ± 12.3	33 ♂ 31 ♀	13.6 ± 3.7	26.0 ± 4.1
Probable	17	13 (77%)	70.8 ± 7.3	9 ♂ 8 ♀	13.6 ± 3.3	26.0 ± 5.7
Possible	21	9 (43%)	61.2 ± 14.0	9 ♂ 12 ♀	12.3 ± 2.2	25.6 ± 4.4
Criteria not fulfilled	26	9 (35%)	64.9 ± 12.5	15 ♂ 11 ♀	14.6 ± 4.8	26.2 ± 2.0

### FDG-PET findings

#### Proportions of likelihood subgroups

According to the matrix defined in section Visual Analysis of FDG-PET we found 32% of all patients fell in the subgroups of high likelihood for PSP/CBD in conjunction with low to moderate likelihood for another neurodegenerative disorder. A high likelihood for PSP/CBD was assigned when there was a pronounced FDG hypometabolism in regions characteristic for PSP (prefrontal cortices, anterior cingulate gyrus, midbrain)/CBD (central region, putamen, thalamus), as illustrated and described in Figure 1. Another 32% of the patients indicated a moderate likelihood for PSP/CBD or equal likelihood for other neurodegenerative disorders, meaning that the hypometabolism pattern was of only moderate severity in the characteristic regions. Thirty-six percent had a low likelihood for PSP/CBD (Table 3).

**Table 3:** Proportions of subgroups (*n* = 117) defined by the likelihood for PSP/CBD and for other neurodegenerative disorders by visual analysis of FDG-PET.

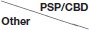	**High (%)**	**Moderate (%)**	**Low (%)**
Low (%)	26	18	18
Moderate (%)	6	7	11
High (%)	1	6	7

#### Overall performance of FDG-PET

Overall performance of binarized FDG-PET (for likelihood PSP/CBD > other ND) for the prediction of a final clinical diagnosis of PSP gave 84% sensitivity, 91% specificity, PPV of 93%, and NPV of 80%. Overall performance of binarized FDG-PET for the prediction of a final clinical diagnosis of suspected CBD was slightly lower, at 71% sensitivity, 76% specificity, PPV of 73%, and NPV of 74%.

#### Influence of clinical probability on FDG-PET findings

The higher clinical probability by diagnostic criteria indicated tendencies toward greater likelihood of PSP/CBD and lower likelihood of other neurodegenerative diseases in FDG-PET. However, statistical testing did not indicate a significance in these probabilities (PSP_CLINIC_ – PSP_FDG_: χ^2^ = 6.6; *p* = 0.16/PSP_CLINIC_ – otherND_FDG_: χ^2^ = 0.7; *p* = 0.95/CBS_CLINIC_ – CBD_FDG_: χ^2^ = 2.4; *p* = 0.66/CBS_CLINIC_ – otherND_FDG_: χ^2^ = 6.4; *p* = 0.17), and positive as well as negative PET scans were found in each clinical category (Table 4).

**Table 4:** FDG-PET likelihoods for PSP/CBD and other ND for all subgroups.

	**Subjects (*n* = 117)**	**FDG-PET likelihood PSP/CBD**			**FDG-PET likelihood other ND**		
		**Low**	**Moderate**	**High**	**Low**	**Moderate**	**High**
Probable PSP	22	5	7	10	13	6	3
Possible PSP	23	9	6	8	15	4	4
PSP criteria not fulfilled	8	5	3	0	5	2	1
Probable CBS	17	4	7	6	9	4	4
Possible CBS	21	8	5	8	17	3	1
CBS criteria not fulfilled	26	11	8	7	13	9	4

#### Overall performance of FDG-PET in combination with clinical parameter

**Figure 2:**
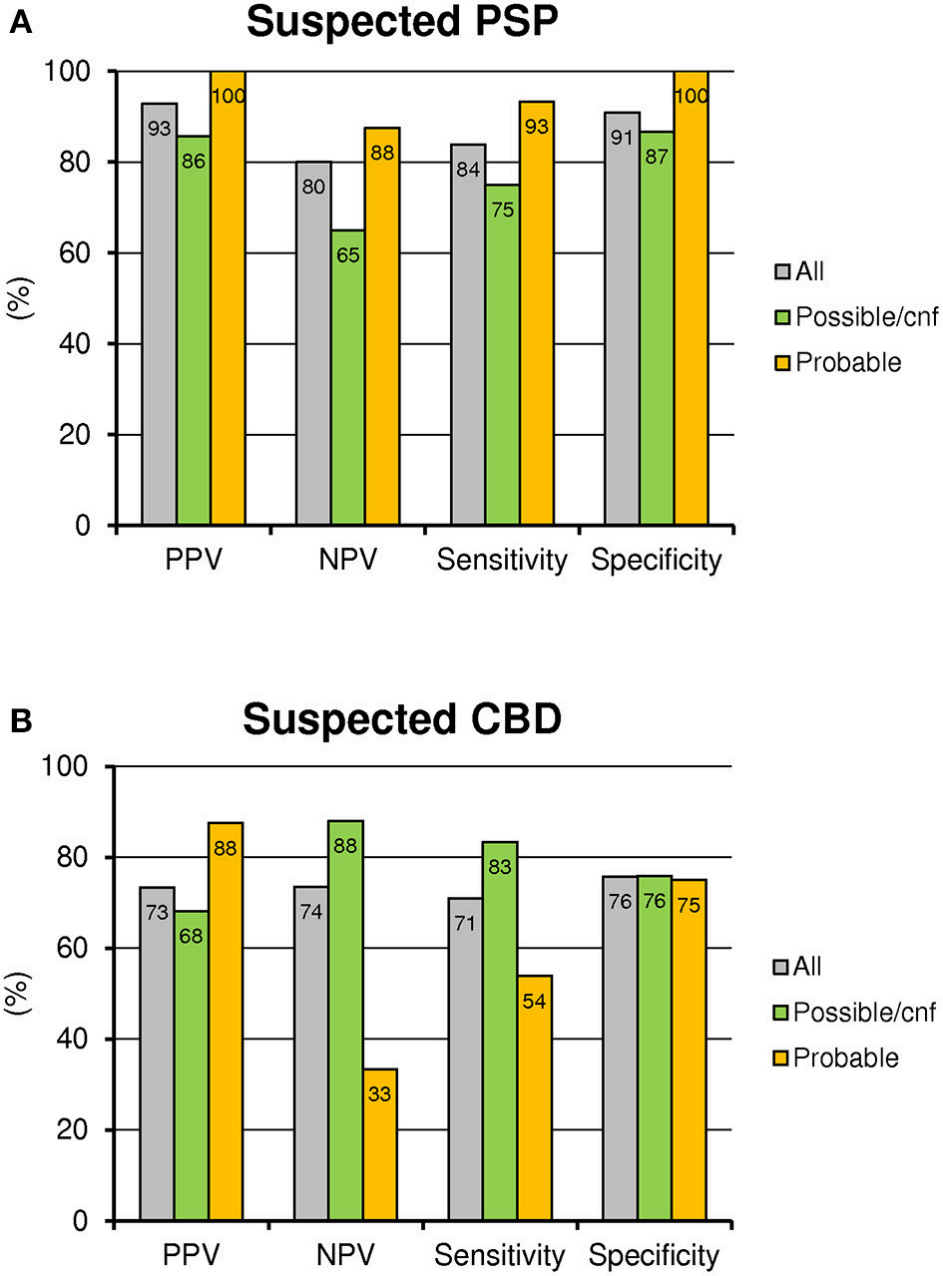
Performance of FDG-PET in clinical subgroups: PPV, NPV, sensitivity and specificity are illustrated for all suspected PSP **(A)** and CBD **(B)** patients and subdivided into possible or incomplete diagnostic criteria (cnf) and probable clinical criteria.

The combined evaluation of clinical probability and binarized FDG-PET for the prediction of the clinical outcome gave discrepant results for suspected PSP and CBD patients. Thus, the suspected PSP patients meeting criteria for probable disease criteria had consistently higher values for PPV, NPV, sensitivity and specificity by FDG-PET when compared to the whole cohort, whereas the suspected PSP patients with only possible or incomplete disease criteria had lower values of these diagnostic accuracy parameters (Figure 2A). Suspected CBD patients meeting probable disease criteria for CBD had a higher PPV but lesser NPV and sensitivity (at equal specificity) by FDG-PET when compared to the whole cohort. Finally, the suspected CBD patients with only possible or incomplete disease criteria showed more sensitivity and higher NPV for FDG-PET when compared to the whole cohort (Figure 2B).

#### Performance of FDG-PET in different likelihood subgroups

**Figure 3:**
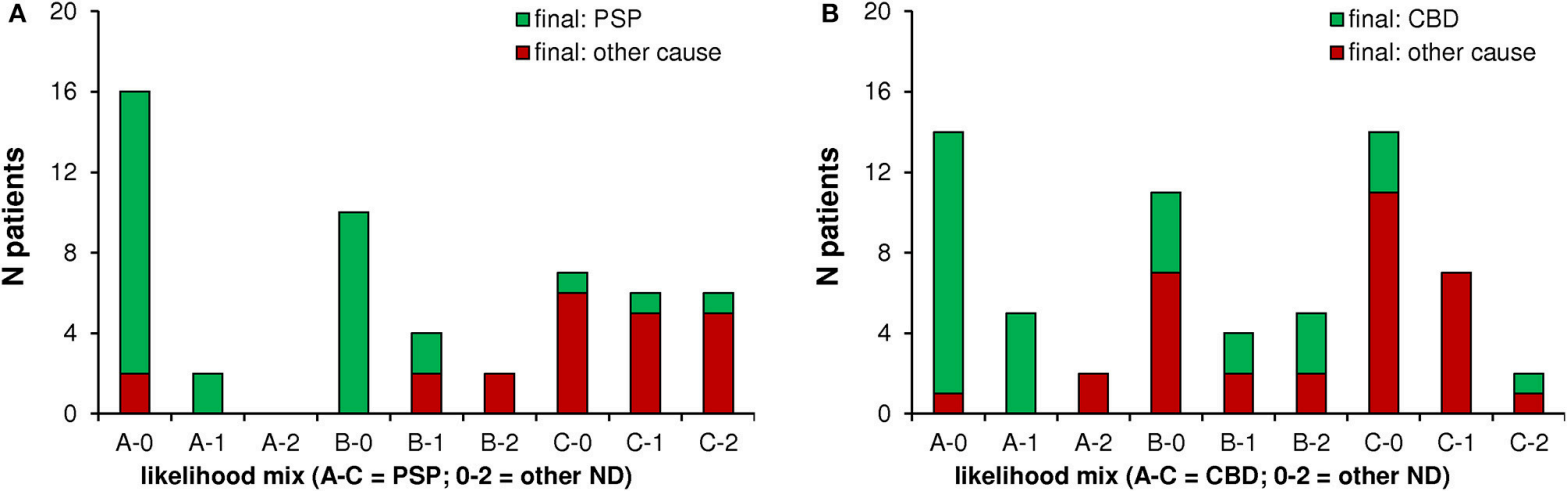
Performance of FDG-PET in imaging based likelihood subgroups: A confirming final diagnosis was frequent when FDG-PET indicated a high likelihood for PSP **(A)**/CBS **(B)** and only a low to moderate likelihood for other neurodegenerative diseases (Groups A-0 and A-1). A final diagnosis of PSP **(A)**/CBS (**B**) was rare when FDG-PET indicated only a low likelihood for a 4R-tauopathy (groups C-0, C-1, and C-2). ND, neurodegenerative disease.

Proportions of patients with a final clinical diagnosis of PSP/CBD differed substantially between the subgroups as defined by FDG-PET (Figure 3). PPV was excellent when FDG-PET indicated a high likelihood for PSP/CBD in combination with low to moderate likelihood for another neurodegenerative disorder (PSP: 95%/CBD: 89%). PPV was distinctly lower when FDG-PET indicated only a moderate likelihood for PSP/CBD or when the likelihood for other neurodegenerative disorders was equal (PSP: 75%/CBD: 41%). FDG-PET had a high NPV for absence of PSP/CBD to clinical follow-up when the initial visual read indicated a low likelihood for PSP/CBD (PSP: 84%/CBD: 83%).

Notably, detection of a metabolism pattern indicative of moderate synaptic dysfunction in brain regions typical for PSP, but no suspicion of other neurodegenerative disorders had a far higher congruency with a positive final clinical diagnosis when compared to the same constellation in patients with suspected CBD (compare subgroups B-0 in Figures 3A,B).

**Figure 4:**
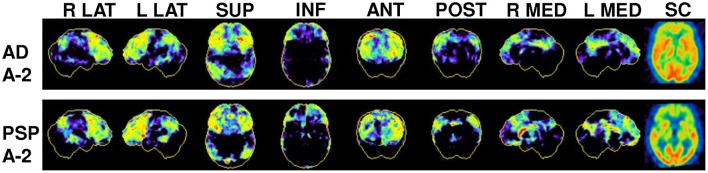
Examples of conflicting FDG-PET findings in two patients with suspected PSP: Representative 3D-SSP for FDG-PET from right/left lateral (R LAT/L LAT), superior (SUP), inferior (INF), anterior (ANT), posterior (POST), right/left medial (R MED/L MED) and a subcortical (SC) horizontal plane image of SUV for comparison of typical AD and PSP metabolic patterns. Note that both patients showed a frontal pronounced hypometabolism but as well an AD-like hypometabolism in the posterior cingulate cortex and in parietal cortices. Clinical follow-up identified one patient as AD and one as PSP.

Binarized analysis of FDG scans revealing a positive pattern for PSP indicated a distinctly higher PPV in the absence of any indication of likelihood for another neurodegenerative disease, when compared to cases with PSP-positive pattern in combination with coexisting likelihood for another neurodegenerative disease (92 vs. 50%). This distinction was less evident for CBD (68 vs. 63%; Figure 4).

### Impact of DaTSCAN results

DaTSCAN (*n* = 49) indicated a dopamine transporter deficit in 65%, a borderline result in 22% and a negative result in 12% of the examined cases. Sixty-six percent of those patients with a pathological DaTSCAN were ultimately diagnosed as PSP/CBS. Of all 28 patients with the final diagnosis PSP/CBS and an available DaTSCAN, 75% showed a pathological result, 21% a borderline result and 4% a negative result. When available, a pathological DaTSCAN together with a pathological FDG-PET (binarized) result for PSP/CBD (*n* = 21) gave a PPV of 76%. Only four patients had overall negative results to the two imaging modalities, which did not support a valid calculation of a NPV. PPV was higher and the NPV was lower for FDG-PET of the (*n* = 68) subjects without an available DaTSCAN compared to all subjects and those with an available DaTSCAN (see Table 5) irrespective of the DaTSCAN result.

**Table 5:** PPV/NPV values of FDG-PET for PSP and CBD for all subjects and divided whether a DaTSCAN was available.

**FDG-PET**	**PPV (%)**	**NPV (%)**
All subjects (*n* = 117)	83	76
DaTSCAN available (*n* = 49)	74	64
No DaTSCAN available (*n* = 68)	90	84

## Discussion

In this clinical study we evaluate FDG-PET in a large dataset of patients with suspicion of PSP or CBD. Previous studies have established the suitability of FDG-PET for the differential diagnosis of PSP and CBD (6, 25, 26). However, most of these investigations were performed in investigational academic settings with a previously selected patient cohort. Studies in nuclear medicine routine settings remain rare and challenging because of more patients with less specific clinical findings and multiple differential diagnoses in the early stage of disease. Therefore, we investigated all subjects referred for a FDG-PET with suspected tau-positive parkinsonism over a period of 7 years. For selected patient cohorts with PSP and/or CBD, the typical FDG-PET patterns have already been demonstrated in group-wise comparisons (5, 7). In clinical routine cases, the confirmation or exclusion of the suspected diagnosis is based on a single PET scan of an individual patient which highlights the importance of a high PPV and NPV for the chosen method.

Our FDG-PET-findings underline the complexity of the diagnostic proceeding for patients with clinically diagnosed parkinsonism with only one third of the patients presenting a high likelihood for PSP/CBD in the visual FDG-PET analysis. Nonetheless, performance of FDG-PET was excellent in those two thirds of the patients, who showed either no clear evidence of a 4R tauopathy pattern (high NPV) or a typical PSP/CBD pattern without conflicting hypometabolism suggestive for other neurodegenerative disorders (high PPV).

However, one third of patients returned inconclusive scans, which gave only low likelihood for a 4R-tauopathy or coexistent hypometabolism patterns typical of other neurodegenerative diseases. Such patients likely benefit from an additional evaluation by more specific PET tracers. Recently developed tau ligands still have some issues that need to be solved, mainly consisting of off-target binding to MAO-B (27). Nonetheless, first human tau PET studies of PSP and CBD already indicated promising results by F-18-THK5351 (18) and F-18-AV1451 (T807) (19, 28, 29). Moreover, new generation tau tracers promise to support a differentiation between 3R and 4R tauopathies. Thus, it seems likely that future tau radioligands will even provide stronger molecular imaging derived differential diagnosis of 4R tauopathies. With regards to cost-effectiveness, the decision to apply such specific but expensive ligands will need to be weighed carefully. Results of the current study suggest that FDG-PET should be performed first in a potential diagnostic algorithm because most clinical suspected diagnosis can be well-supported by a specific FDG-PET pattern at high PPV and NPV. Although unspecific for different protein depositions, FDG-PET offers an advantage as primary molecular imaging method because of the potential detection of tau-negative neurodegenerative disorders through their distinct hypometabolism pattern. This is highly relevant in cohorts of suspected PSP and CBS patients, as tau-negative variants of fronto-temporal dementia and multiple system atrophy are potential differential diagnoses. Based on our current analysis, FDG-PET can also identify subjects who need further evaluation with additional molecular imaging such as tau-PET imaging. The current dataset creates the basis for future prospective trials evaluating diagnostic algorithms and the additive value of tau-PET in PSP and CBD.

FDG-PET performed better in patients with suspected PSP than for patients with suspected CBD probably due to a more difficult clinical diagnosis (30, 31) and a FDG-pattern less distinct from other neurodegenerative diseases (6). In conclusion, suspected CBD patients with conflicting clinical findings might even profit from an evaluation by a more specific biomarker such as tau radioligands prior to FDG-PET. However, since newest PSP criteria now consider non PSP-RS entities as well, the situation for PSP could as well get more complex, with an assumed benefit by imaging techniques (11). Dual PET tracer studies with randomized sequence will be needed to answer this question.

Our data indicate that clinical probability should be considered when interpreting a FDG-PET of PSP or CBD patients and when planning further evaluation. For PSP patients with a probable clinical likelihood, the higher overall performance of the method needs to be considered in comparison with patients that have only possible or absent diagnostic criteria. Especially the low NPV of a negative FDG-PET read out in the subcohort of suspected PSP patients with possible or absent diagnostic criteria has to be stressed. This subgroup should benefit from further evaluation, where tracers with higher sensitivity are demanded.

The situation in suspected CBD patients presented even more complex: First, CBD patients with a probable clinical likelihood had a very high PPV by a positive FDG-PET, which implies that positive scans of such patients lead to a very high diagnostic confidence. On the other hand, we observed only a very low NPV by a negative FDG-PET in this subcohort. Thus, negative FDG-PET results of CBD patients with high clinical likelihood have only a low meaningfulness and further evaluation by a more sensitive tracer will definitely improve the diagnostic accuracy in this subgroup. Furthermore the PPV of a positive FDG-PET in the subgroup of CBD patients with possible or absent diagnostic criteria was only moderate and indicated another potential target group for beneficial further evaluation.

For the detection of PSP, automatic analysis strategies such as machine learning algorithms revealed a slightly lower PPV (91 vs. 93%) but a noticeable higher NPV (92 vs. 80%) than we observed in our sample (14). This comparison should be interpreted with caution due to the differing population characteristics. Nevertheless, the majority of clinical nuclear medicine departments still use visual inspection of axial slices and surface projection Z-score maps for FDG-PET of patients vs. in house norm collectives. Although one third of patients did not show distinct visual FDG-PET patterns to confirm the diagnosis, the diagnostic modality helps the nuclear medicine clinician to decide upon a rational recommendation for further evaluation of the patient.

Dopamine transporter availability was only performed in less than half of the patients (*n* = 49/117). This was probably related to certainty of clinicians about the present parkinsonism in most of the analyzed patients. Interestingly, the PPV for combined pathological DaTSCAN and FDG-PET was lower than for patients with only a pathological FDG-PET. Moreover, patients without an available DaTSCAN showed a higher PPV for detecting PSP/CBD by FDG-PET compared to those with an available DaTSCAN. This indicates the unfavorable consequence when clinicians order an additional DaTSCAN if the clinical presentation is inconclusive, which results in a lower pretest probability, thus presenting a greater diagnostic challenge for FDG-PET. Due to the limited number of cases with an available DaTSCAN, we cannot test if dopamine transporter SPECT would have facilitated the establishment of a correct differential diagnosis in cases inconclusive to FDG-PET alone.

## Limitations

The impact of MRI results on the diagnostic accuracy has not been investigated in this setting. In the present workup, patients with clear findings in MRI were normally not referred for molecular imaging by FDG-PET. Thus, we did not assume an increasing value of analyzing inhomogeneous MRI findings which do not reflect the patient cohort initially referred to MRI. It should be noted that this cohort of clinical routine imaging in nuclear medicine at a tertiary center definitely underwent preselection steps before going to FDG-PET. Thus, the present investigation potentially presents more complex patients when compared to PSP or CBD patients referred to a primary care center. Final diagnoses were based on clinical follow-up ≥ 1 year without histopathological validation and thus no gold standard was available as “true” diagnosis. Furthermore, imaging findings of FDG-PET certainly contributed to the initial establishment of a clinical diagnosis, especially in cases that did not fulfill the diagnosis criteria completely. Therefore, some degree of circular interference cannot be completely ruled out in such a clinical dataset, but the verification of all single items within the retrospective survey strengthened the validity of clinical diagnoses.

## Conclusions

We investigated clinical routine cases with the differential diagnosis of PSP/CBD referred for a FDG-PET to reveal the diagnostic value for the final diagnosis. FDG-PET showed a high PPV for subjects with a typical PSP/CBD pattern and a low likelihood for another ND as well as a high NPV for subjects without a typical PSP/CBD pattern. One third of patients (CBD > PSP) showed inconclusive scans and might profit from further evaluation with more specific radioligands (e.g., tau), where FDG-PET can act as a gatekeeper for such more expensive methods. This subgroup represents an ideal cohort for testing the additive value of tau radioligands in prospective trials for 4R tauopathies. Clinical probability by diagnostic criteria should be considered together with FDG-PET findings when evaluating the benefit of scanning with additional ligands.

## Ethics statement

Retrospective analysis of PET data was approved by the local ethics committee of the LMU Munich (399-09).

## Author contributions

LB: conception and design, acquisition of data, analysis and interpretation of data, document writing and editing. JM-W: acquisition of data, analysis and interpretation of data, document writing. JS, EB, MU, NA, and PB: interpretation of molecular imaging data, acquisition of data, revising of the manuscript. SS, CP, GN, OP, RP, CC, KBu, and KBö: acquisition of patient data, drafting and revising of the manuscript. JL: acquisition of patient data, conception and intellectual input, drafting and revising of the manuscript. AR: interpretation of molecular imaging data, conception and intellectual input, drafting and revising of the manuscript. MB: conception and design, acquisition of data, document editing, final manuscript approval for submission and publication.

### Conflict of interest statement

PB declares a research collaboration with GE. AR received speaker honoraria from Piramal Imaging and GE Healthcare. The remaining authors declare that the research was conducted in the absence of any commercial or financial relationships that could be construed as a potential conflict of interest.
