# Supplementary material for: Clinical Routine FDG-PET Imaging of Suspected Progressive Supranuclear Palsy and Corticobasal Degeneration: A Gatekeeper for Subsequent Tau-PET Imaging?
Source: Front Neurol. 2018 Jun 20;9:483. doi: 10.3389/fneur.2018.00483 (PMC6019471; doi:10.3389/fneur.2018.00483)
Supplement: Supplementary file 1 [file Table_1.DOCX]

**Supplemental Methods**

Patients were classified for probable or possible PSP according to NINDS-SPSP criteria ([Litvan, Agid et al. 1996](#_ENREF_15)):

|  | **Mandatory inclusion criteria** | **Supportive criteria** |
| --- | --- | --- |
| **Probable** | 1. Gradually progressive disorder 2. Onset at age 40 or later 3. Vertical (upward or downward gaze) supranuclear palsy and prominent postural instability with falls in the first year of disease onset 4. No evidence of other diseases that could explain the foregoing features | - Symmetric akinesia or rigidity, proximal more than distal - Abnormal neck posture, especially *retrocollis* - Poor or absent response of parkinsonism to levodopa therapy - Early onset of cognitive impairment |
| **Possible** | (1), (2), (4)   1. Either vertical (upward or downward gaze) supranuclear palsy **or** both slowing of vertical saccades and prominent postural instability with falls in the first year of disease onset |  |

***Supplemental Table 1:*** *Condensed version of the NINDS-SPSP criteria for the clinical diagnosis of probable and possible PSP.*

Patients were classified for probable or possible CBS according to criteria defined by Armstrong et al ([Armstrong, Litvan et al. 2013](#_ENREF_2)):

|  | **Clinical research criteria** |
| --- | --- |
| **Probable** | 1. Insidious onset and gradual progression 2. Minimum duration of symptoms: one year 3. Onset at age 50 or later 4. Asymmetric presentation of two of (a) limb rigidity or akinesia (b) limb dystonia (c) limb myoclonus plus two of (d) orobuccal or limb apraxia (e) cortical sensory deficit (f) alien limb phenomena |
| **Possible** | (1), (2)   1. No minimum for age at onset 2. Possible symmetric presentation of one of (a-c) plus one of (d-f) |

***Supplemental Table 2:*** *Condensed version of the criteria for the diagnosis of probable and possible corticobasal degeneration defined by Armstrong et al.*

**Supplemental Results**

| **Deficits** | **Suspected PSP**  **(n=53)** | **Suspected CBD**  **(n=64)** |
| --- | --- | --- |
| **Motoric** | | |
| Vertical supranuclear palsy | 68% | 11% |
| Slowing of vertical saccades and postural instability | 47% | 9% |
| Vertical supranuclear palsy and prominent postural instability with falls | 38% | 2% |
| Akinetic rigid syndrome | 27% | 42% |
| Symmetric akinesia or rigidity | 21% | 5% |
| Dystonia | 10% | 29% |
| Limb apraxia | 4% | 64% |
| Abnormally position of head/ neck | 2% | 3% |
| Myoclonus | 2% | 13% |
| Alien limb syndrome | 0% | 16% |
| Dysphagia/ dysarthria | 23% | 13% |
| Levodopa response | 33% | 23% |
| **Sensory** | | |
| Cortical sensory loss | 2% | 42% |
| Visuospatial deficits | 10% | 8% |
| **Cognitive** | | |
| Speech and language impairment | 31% | 34% |
| Frontal executive dysfunction | 10% | 11% |
| **CERAD**   - Verbal fluency (Animals) - Modified BNT - Word List Learning - Constructional Praxis - Word List Recall - Word List Recognition-Discriminability (%) - Verbal fluency (S-Words) - TMT-A (sec) - TMT-B (sec) | **64.7 ± 17.8**  8.7 ± 4.6  10.1 ± 5.8  17.0 ± 4.7  18.2 ± 9.0  4.8 ± 2.4  94.5 ± 10.2  10.1 ± 5.8  47.9 ± 51.6  116.9 ± 57.2 | **70.8 ± 18.1**  9.2 ± 2.8  9.8 ± 4.2  18.1 ± 4.3  23.1 ± 8.3  5.3 ± 2.6  94.6 ± 7.3  14.4 ± 5.6  26.0 ± 26.6  92.5 ± 68.9 |

***Supplemental Table 3:*** *Frequency of clinical symptoms and parameter of neuropsychological including motoric, sensory and cognitive categories for subjects with suspected PSP/CBD.*
